# Multiple Fatalities in a Family Cluster of COVID-19 With Acute Respiratory Distress Syndrome

**DOI:** 10.31486/toj.20.0056

**Published:** 2020

**Authors:** Adam Lowe, Donald D. Chang, Grady Creek

**Affiliations:** ^1^ The University of Queensland Faculty of Medicine, Ochsner Clinical School, New Orleans, LA; ^2^ Section of Intensive Care, Department of Pulmonary Diseases, Ochsner Baptist, New Orleans, LA

**Keywords:** *Comorbidity*, *coronavirus*, *genetic predisposition to disease*, *respiratory distress syndrome–adult*, *risk factors*, *SARS-CoV-2*

## Abstract

**Background:** Cases of COVID-19 family clusters have been reported across the globe. While disease severity can vary widely, reports of severe infection leading to multiple fatalities within a family are limited.

**Case Report:** Four family members each presented to the emergency department with fever and upper respiratory symptoms. Each individual tested positive for severe acute respiratory syndrome coronavirus 2 (SARS-CoV-2) infection via nasopharyngeal swab. All individuals developed acute respiratory distress syndrome refractory to conventional medical therapy and subsequently died from their disease.

**Conclusion:** This report describes a familial cluster of fatal COVID-19 infections and suggests a potential genetic predisposition for severe disease, emphasizing the importance of investigating family clusters of severe COVID-19 infection to determine host and viral factors that may predispose to a severe disease course. Such investigations could improve our understanding of the disease and guide preventive measures for at-risk populations.

## INTRODUCTION

In December 2019, the Wuhan Municipal Health Commission reported the first cases of severe acute respiratory syndrome coronavirus 2 (SARS-CoV-2) in China, with the associated clinical manifestation termed COVID-19 (coronavirus disease 2019) that has since caused a global pandemic.^1,2^ Two family clusters were noted among these initial cases, with one cluster demonstrating evidence of person-to-person transmission.^1^ Since then, cases of family clusters have been reported worldwide, with disease severity ranging from mild flu-like symptoms to fatal acute respiratory failure.^3^ Preexisting comorbidities, sex, and race have been reported as risk factors for severe COVID-19.^4-6^ The evidence base for a familial predisposition toward severe complications secondary to COVID-19 is growing, as underscored by a New Jersey family cluster with multiple fatalities from COVID-19.^7^ However, the number of published reports of family clusters of COVID-19 infections resulting in fatal respiratory disease is sparse.^8^

We describe a family cluster of 4 COVID-19–positive patients who all developed acute respiratory distress syndrome (ARDS) that ultimately led to death.

## CASE SERIES

In early March 2020, 4 family members living in the same household were hospitalized with fever and respiratory symptoms. None of the family members had a history of travel. Patient 1, the matriarch and index case in the family, reported possible exposures from caregivers who exhibited flu-like symptoms or from attending church services.

### Patient 1

**Figure 1.**
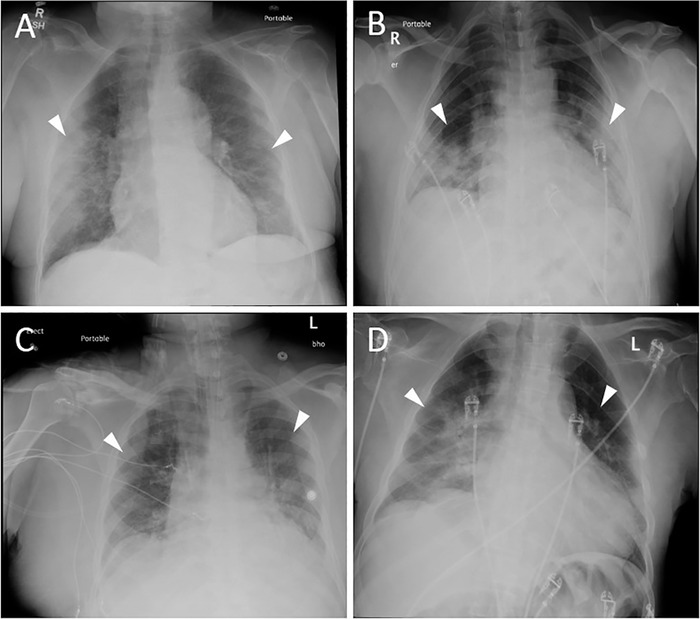
**Chest x-ray for (A) 86-year-old mother 2 days after admission shows bilateral ground-glass opacities (white arrowheads), more severe on the right. Chest x-rays for (B) the 58-year-old son, (C) the 71-year-old son, and (D) the 61-year-old son all displayed bilateral airspace opacities on emergency department presentation (white arrowheads).**

An 86-year-old African American female with a history of adequately controlled diabetes mellitus (HbA1c 6.4%), coronary artery disease, hypertension, and Alzheimer disease presented on March 8 for symptoms of fever, vomiting, and shortness of breath for 24 hours and a syncopal episode 1 day prior to symptom onset. In the emergency department (ED), her chest x-ray was unremarkable, and influenza assay was negative. She was febrile at 102.4°F in the ED and was admitted for observation. On March 10, the patient developed worsening dyspnea and was placed on 2 L of supplemental oxygen via nasal cannula. Repeat chest x-ray showed bilateral ground-glass opacities, more severe on the right (Figure 1A). C-reactive protein was 155.9 mg/dL. Nasopharyngeal swab submitted for SARS-CoV-2 qualitative polymerase chain reaction (PCR) testing returned positive. The patient developed respiratory failure and was transferred to the intensive care unit (ICU) on March 12. She was supported with noninvasive ventilation. Urine antigen for *Streptococcus pneumoniae* collected March 12 tested positive, and the patient was started on methylprednisolone (60 mg intravenous [IV] daily), azithromycin (500 mg IV daily), ceftriaxone (1 g IV daily), and hydroxychloroquine (800 mg loading dose, followed by 400 mg daily). On March 16, her respiratory status improved, and she was transferred out of the ICU. On March 18 (day 12 of symptoms), the patient again developed worsening dyspnea and hypoxemia with oxygen saturation of 86% with a non-rebreather mask. The family made the decision to transition the patient to home hospice care. She was discharged on March 22 and died in her home on March 23.

**Figure 2.**
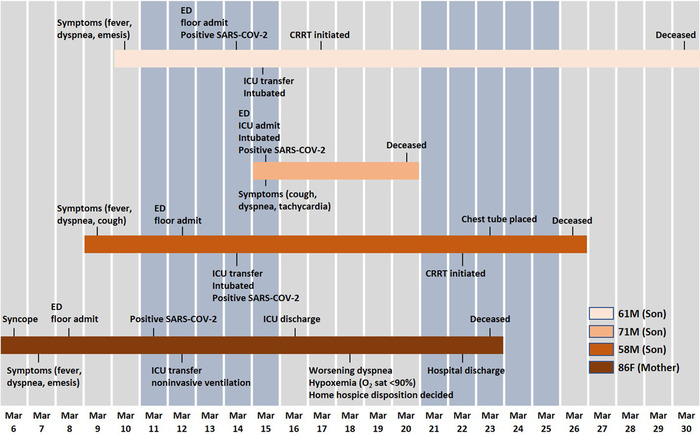
**Timeline of exposure and symptoms of the family cluster—a mother and 3 sons—with COVID-19.** CRRT, continuous renal replacement therapy; ED, emergency department; F, female; ICU, intensive care unit; M, male; O_2_ sat, oxygen saturation; SARS-CoV-2, severe acute respiratory syndrome coronavirus 2.

### Patient 2

The 58-year-old son of Patient 1 also had a history of adequately controlled diabetes mellitus (HbA1c 6.5%) and presented to the ED on March 12 for 3 days of cough, shortness of breath, and fever. In the ED, he had a fever of 102.2°F and hypoxemia, with an oxygen saturation of 90% on room air. Influenza assay was negative. Chest x-ray showed bilateral airspace opacities (Figure 1B). Nasopharyngeal swab submitted for SARS-CoV-2 PCR testing returned positive. The patient was admitted and started on ceftriaxone (1 g IV daily), azithromycin (500 mg IV daily), and hydroxychloroquine (800 mg loading dose, followed by 400 mg daily). On March 14 (day 5 of symptoms), his hypoxemia worsened despite a non-rebreather mask, with oxygen saturation decreasing to as low as 70%. C-reactive protein was 142 mg/L, and ferritin was 2,188 ng/mL. He was transferred to the ICU where he was intubated and mechanically ventilated. The patient met criteria for severe ARDS with an arterial oxygen partial pressure (PaO_2_)/fraction of inspired oxygen (FiO_2_) ratio as low as 98. He was treated with a protocol of daily prone positioning. The ICU used a protocol for prone positioning similar to that used in the Effect of Prone Positioning on Mortality in Patients With Severe and Persistent Acute Respiratory Distress Syndrome (PROSEVA) study.^9^ On March 22, he developed acute kidney injury (AKI) requiring initiation of continuous renal replacement therapy (CRRT). Medical record review revealed no history of chronic kidney disease (CKD). Because of clotting of the dialysis circuit, he was treated with a continuous infusion of unfractionated heparin. On March 23, he developed bilateral pneumothoraces requiring chest tube placement. His condition deteriorated despite these measures. The patient developed shock refractory to vasopressor therapy and died on March 26.

### Patient 3

The 71-year-old son of Patient 1 with a history of hypertension, CKD (stage 3), and sarcoidosis (inactive disease, not on treatment) presented to the ED on March 15. Symptoms included cough, fever of 102.6°F, and shortness of breath. He was tachycardic and hypoxemic, with an oxygen saturation as low as 70% on room air. Influenza assay was negative. Chest x-ray showed bilateral airspace opacities (Figure 1C). C-reactive protein was 90.6 mg/L. Nasopharyngeal swab submitted for SARS-CoV-2 PCR testing returned positive. The patient was admitted directly to the ICU where he was intubated and mechanically ventilated. He met criteria for severe ARDS with a PaO_2_/FiO_2_ ratio as low as 76. He was treated with a protocol of daily prone positioning. Medications included ceftriaxone (1 g IV twice daily) and hydroxychloroquine (800 mg loading dose, followed by 400 mg daily). The patient's condition deteriorated rapidly, and he died on March 20.

### Patient 4

The 61-year-old son of Patient 1 with a history of hypertension, multiple sclerosis, and seizure disorder presented to the ED on March 14 with symptoms of fever, vomiting, and shortness of breath for 4 days. He was hypoxemic with an oxygen saturation of 90% on room air and febrile at 101.3°F upon presentation. Chest x-ray showed bilateral airspace opacities, more severe on the right (Figure 1D). Influenza assay was negative. Nasopharyngeal swab submitted for SARS-CoV-2 PCR testing returned positive. On March 15 (day 5 of symptoms), the patient's hypoxemia worsened, and he was moved to the ICU where he was intubated and mechanically ventilated. C-reactive protein was 338 mg/dL, and lactate dehydrogenase was 714 U/L. He met criteria for severe ARDS with a PaO_2_/FiO_2_ ratio of 73. He was treated with a protocol of daily prone positioning. He developed AKI requiring initiation of CRRT on March 17. Medical record review revealed no history of CKD. Because of clotting of the dialysis circuit, he was treated with a continuous infusion of unfractionated heparin. Medications included azithromycin (500 mg IV daily), ceftriaxone (1 g IV daily), hydroxychloroquine (800 mg loading dose, followed by 400 mg daily), and dexamethasone (20 mg IV daily). Despite attempts to wean the patient off mechanical ventilation, he was unable to support his own respiratory efforts and remained on ventilation throughout his ICU stay. On March 30, the patient developed shock refractory to vasopressor therapy and died.

## DISCUSSION

Multiple members of a family concurrently developing COVID-19 infections from the virus is not surprising. However, multiple members developing fatal disease should give pause. As of early May 2020, the observed case-fatality rate in the United States was 5.9%,^10^ but the actual case-fatality rate was thought to be lower.^11^ The family cluster reported here is an anomaly (Figure 2). A similar occurrence was reported in New Jersey where COVID-19 infection took the lives of 4 family members.^7^ We suspect other family clusters of severe disease will emerge as COVID-19 infections are identified globally.

From the onset of the COVID-19 pandemic, clinicians have struggled to understand why some infected patients experience only mild symptoms while others exhibit progressive, fatal disease. Inheritable traits that predispose to severe complications of COVID-19 infection may exist. Family clusters, such as this one, could be a key to identifying host factors that predispose to a severe disease course and to understanding why subsets of our population are at increased risk. Louisiana data as of April 2020 show hypertension as the leading comorbid condition associated with death in patients with COVID-19, followed by diabetes.^12,13^ The Centers for Disease Control and Prevention (CDC) has identified both hypertension and diabetes as risk factors associated with severe disease,^14^ and both conditions were present in this family cluster. Age is another significant risk factor for mortality, with data from the CDC showing a consistent rise in mortality after the age of 40 years and doubling each decade after the age of 50 years.^15^ All members of this family were older than 50 years. In addition, all members were African American. In Louisiana, more than 70% of COVID-19–related deaths have occurred among African Americans who represent slightly more than 30% of the state's population.^13^

Another important consideration is how viral mutations can manifest within a family cluster. Other previously studied coronaviruses have demonstrated high mutation rates.^16^ As a result, a more pathogenic sublineage of the virus could emerge and manifest within a family by causing severe disease. As researchers endeavor to trace signature mutations in the viral genome sequence,^17^ including viral samples from family clusters such as this may prove informative.

## CONCLUSION

This report provides evidence of the need for further investigation into family clusters of severe COVID-19 infection to identify both host and viral factors that may predispose to a severe disease course. Such investigations could improve our understanding of the disease and guide preventive measures for at-risk populations.

## Notes

This article meets the Accreditation Council for Graduate Medical Education and the American Board of Medical Specialties Maintenance of Certification competencies for Patient Care and Medical Knowledge.
